# Band-Gap Energies of Choline Chloride and Triphenylmethylphosphoniumbromide-Based Systems

**DOI:** 10.3390/molecules25071495

**Published:** 2020-03-25

**Authors:** Alberto Mannu, Maria Enrica Di Pietro, Andrea Mele

**Affiliations:** 1 Department of Chemistry, Materials and Chemical Engineering “G. Natta”, Politecnico di Milano, Piazza L. da Vinci 32, 20133 Milano, Italy; mariaenrica.dipietro@polimi.it (M.E.D.P.);; 2 CNR-SCITEC Istituto di Scienze e Tecnologie Chimiche, Via Alfonso Corti 12, 20133 Milano, Italy

**Keywords:** deep eutectic solvents, deep band-gap systems, UV–VIS, Tauc plot, molten systems

## Abstract

UV–VIS spectroscopy analysis of six mixtures containing choline chloride or triphenylmethylphosphonium bromide as the hydrogen bond acceptor (HBA) and different hydrogen bond donors (HBDs, nickel sulphate, imidazole, d-glucose, ethylene glycol, and glycerol) allowed to determine the indirect and direct band-gap energies through the Tauc plot method. Band-gap energies were compared to those relative to known choline chloride-containing deep band-gap systems. The measurements reported here confirmed the tendency of alcohols or Lewis acids to increment band-gap energy when employed as HBDs. Indirect band-gap energy of 3.74 eV was obtained in the case of the triphenylmethylphosphonium bromide/ethylene glycol system, which represents the smallest transition energy ever reported to date for such kind of systems.

## 1. Introduction

In recent years, the interest toward organic molten systems showing a eutectic point has considerably increased. When some hydrogen bond donors (HBDs) and acceptors (HBAs) are combined in opportune proportions, the resulting mixture shows an important drop in melting point (with respect to the parent pure constituents), high viscosity, low volatility, and good solvent properties. These organic mixtures are named deep eutectic systems or, sometimes, deep eutectic solvents (DESs) and are exploitable in several fields. To cite only some applications of DESs, they have been proposed as new solvents [1,2,3,4], catalysts [5], electrodeposition agents [6,7], and stabilizing agents for antibiotics [8].

Since the first studies by Abbott [9], consistent research activity has been devoted to the structure–activity relationship of such systems. In fact, the specific network of hydrogen bonds, decorated by holes and charges, which exists only for specific combinations of HBDs and HBAs, determines the peculiar chemical–physical properties of DESs and DES-like systems [10].

Recently, we added band-gap energy to the physical descriptors that can be used for DES characterization. Some mixtures of HBDs and HBAs also show a drop in band-gap (BG) energy at the eutectic composition [11]. Due to this peculiar characteristic, these systems are named deep band-gap systems (DEBAGs). It has been observed that DEBAGs also act as DESs, as the specific molar ratio between HBA and HBD showing the drop in BG energy is the same as that showing a drop in melting point [1]. Not all DESs are DEBAGs, as in the case of choline chloride/levulinic acid, choline acetate/glycolic acid, and choline acetate/ethylene glycol systems [1]. 

Drops in melting point and BG energy are related to the specific chemical structure of these organic molten salts. In these mixtures, local density fluctuations generate empty spaces that are filled by charges moving all around the hydrogen bond network. The model of Fürth [12] has been employed to describe such systems and to calculate some structural parameters, such as hole size [13]. The same model can explain the peculiar optical characteristics of such systems. In fact, it has been proposed that, in analogy to the change in the fluorescent emission of some protein amyloids [14], the lowering variation of BG energy in eutectic molten salts can be related to charge delocalization through hydrogen bonding, which indicates a proton-transfer mechanism through hydrogen bonds, causing a variation in the optical properties of the system [1]. Depending on the nature of the HBAs and HBDs, different ionic H-bonds can be formed with different strength and geometry [15,16]. Thus, the possibility of tuning the properties of eutectic systems by acting on their components in terms of type and/or molar fraction is of particular interest. 

A relevant advantage of DEBAGs compared to DESs is represented by the possibility of easily and quickly determining lower band-gap composition by UV–VIS spectroscopy. From spectroscopic data, BG energy can be graphically extrapolated by employing the Tauc plot method [17]. In the case of DESs, in order to determine the eutectic composition for a HBA and HBD mixture, a phase diagram should be built, which can be a difficult task. 

The approach based on tuning BG energy in organic systems by mixing components in different proportions was already presented as innovative for the development of organic semiconductors [18]. In this context, the search for mixtures of specific HBAs and HBDs that show BG energy that is as small as possible is an important task. Currently, few DEBAGs have been described, and more examples are needed in order to gain more information about their behavior and characteristics. 

Herein, six systems containing choline chloride as the HBA and different HBDs (nickel sulphate (**1**), imidazole (**2**), d-glucose (**3**), glycerol (**4**)), or triphenylmethylphosphonium bromide as the HBA and ethylene glycol (**5**) or glycerol (**6**) as HBDs are described in terms of band-gap energy. Due to the very high viscosity of Systems **1**–**3** at room temperature, 10 wt % of water was added after melting and prior to UV–VIS measurements. From the UV–VIS data, optical-transition energy was determined by the Tauc plot method. The full procedure is described, and the results are discussed and compared with the literature data referring to similar systems.

## 2. Results and Discussion

Six eutectic systems were considered in the present study. Table 1 reports the nomenclature and composition.

Systems containing choline chloride/imidazole (**2**) [19], glucose (**3**) [20], or glycerol (**4**) [20], and triphenylmethylphosphonium bromide/ethylene glycol (**5**) [21] or glycerol (**6**) [21] have already been described as DESs. The system composed by choline chloride and nickel sulphate was prepared in analogy to the parent choline chloride/zinc chloride [9] and choline chloride/copper chloride [1,22] DEBAGs. 

10 wt % of water was added to systems **1**–**4**, and the corresponding solutions were analyzed by UV–VIS spectroscopy. 

**Figure 1:**
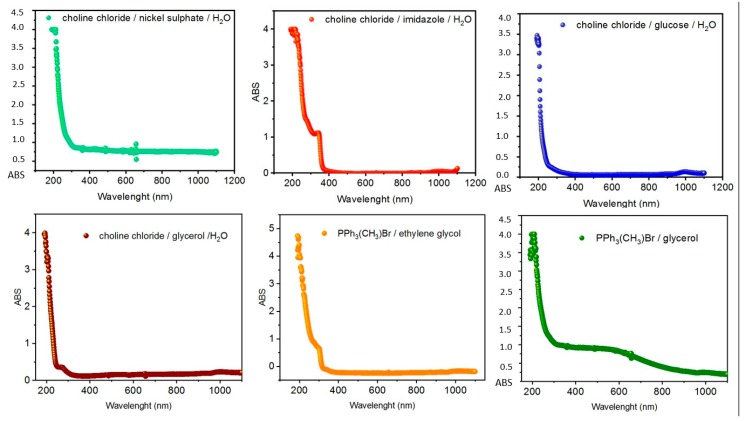
UV–VIS absorbance plots for (top-left) **1**, (top-center) **2**, (top-right) **3**, (bottom-left) **4**, (bottom-center) **5**, and (bottom-right) **6**.

The corresponding absorbance plots are reported in Figure 1.

Looking at the plots reported in Figure 1, absorbance peaks are appreciable only for Systems **2**, **4**, and **5**, while they are absent for Systems **1**, **3**, and **6**. This absence of relevant peaks between 400 and 800 nm was exploited formany DESs employed as solvents for the UV–VIS quantification of analytes such as lignin [23] or gold nanoparticle [24]. 

Nevertheless, UV–VIS spectra of molten salts hide valuable information that can be extracted with the appropriate methodology. The Tauc method allows to calculate the band-gap energy from acquired UV–VIS data [25]. In order to implement the Tauc plot, it is necessary to calculate the optical-absorbance-coefficient values and the photon energy as follows. 

Calculation of photon energy from the Tauc and Davis–Mott Relation [26]:

(αην)^n^ = k (ην − Eg), (1)

where α = absorption coefficient, ην = incident photon energy, k = energy-independent constant, Eg = optical band gap, and n = nature of the transition (n = 2 for direct and ½ for indirect). 

Conversion of wavelength to energy and calculation of absorbance coefficient α:

Eg = hν (2)

Max Planck equation (h = Planck constant; ν = incident photon):

ν = c/λ (3)

where c = speed of light, λ = wavelength of incident photon; ν was used instead of the usual n to distinguish it from n = nature of the transition.

Eg = hc/λ,(4)

where h = 6.62E-34 Js; c = 2.999E8 m/s; Eg = (19.85E-26 Jm)/λ.

After the conversion from J to eV, the following equation can be obtained: Eg = 1239.3 nm/λ (Energy).

The value of Eg was employed to convert UV–VIS data acquired through direct measurements into energy values for the X axis, employing the formula

X = Eg/absorbance(5)

The absorbance coefficient is calculated as follows [27]

(6) $$I/I_{0}=e^{-\alpha l},$$

where I = intensity of transmitted light; I_0_ = intensity of incident light; α = absorbance coefficient; l = path length.

From Equation (6) the following absorbance coefficient can be obtained: α = 2.303 A cm^−1^

α was used to obtain the values for the Y axis of the Tauc plot, through the formula

(7) $$Y=\sqrt{2.303\times \;\mathrm{absorbance}\;\times \;X}$$

for the indirect band gap, and

Y = (2.303 × absorbance × X)^2^ (8)

for the direct band gap.

**Figure 2:**
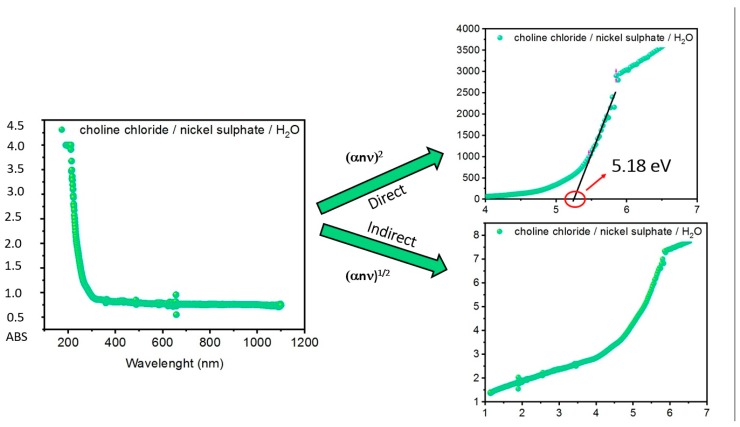
UV–VIS spectrum and Tauc plots for indirect and direct transition of System **1**.

By employing Equations (5) and (7) or (8), it is possible to obtain for each system analyzed by UV–VIS the plots corresponding to the indirect (Equation (7)) and direct (Equation (8)) transition, as reported in Figure 2 for System **1** (choline chloride/nickel sulphate/H_2_O). 

The plots for both transitions reported in Figure 2 were used for graphically determining the nature of the transition. For each plot, if a linear part of the curve was available, it was possible to calculate the energy gap by applying a linear fitting equation (y = a + bx) and intercepting the X axis in the point corresponding to the energy gap (eV). As a matter of fact, the choice of the linear part of the curve to be fitted is arbitrary and considering different ranges can result in a slight change in the final estimated energy-gap value. In order to minimize the error associated with this procedure, we finally considered the linear part of the curve with a Pearson’s r value > 0.95 (Table 2). 

For System **1**, a direct band-gap energy of 5.18 eV could be estimated (Figure 2, top-right), while the curve corresponding to the indirect transition was not suitable for determining a band-gap value, suggesting that an indirect transition was not allowed.

**Figure 3:**
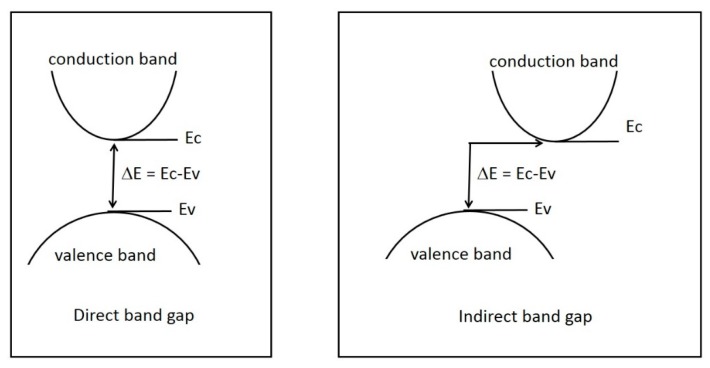
Schematic and simplified representation of direct and indirect optical transition.

In insulators and semiconductors, band-gap energy corresponds to the minimal amount of energy required to promote the transition of one electron from the valence band to the conduction band. The energies of the two former bands are characterized by a specific crystal momentum that is represented by a k-vector. When the two k-vectors have the same value, the top of the valence band and the bottom of the conduction band are aligned in the k-space (space of wave vector), and the band gap is direct (Figure 3, left). In this case, photon absorption is necessary for electron transition from the valence band to the conduction band. When k-vectors are different, the top of the valence band and the bottom of the conduction band were not aligned in the k-space (Figure 3, right), and the additional participation of a phonon that would change the momentum of the electron was necessary for the transition to happen [17,28].

**Figure 4:**
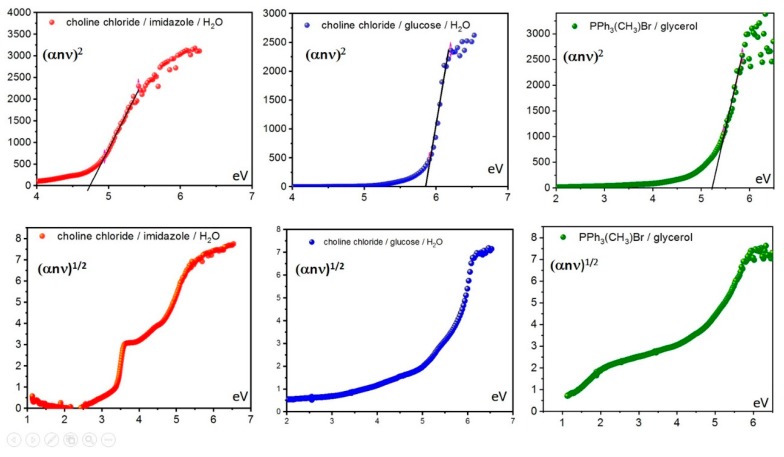
Tauc plots for (top) direct and (bottom) indirect transition of Systems (left) **2**, (center) **3**, and (right) **6**.

The above-described protocol was also applied to Systems **2**–**6**, revealing some interesting information. Similarly to System **1**, obtained curves for Systems **2**, **3**, and **6** showed no consistent linear part when indirect band-gap determination was attempted, thus revealing only direct transition, with a relative band gap of 4.74, 5.85, and 5.23 eV, respectively (Figure 4). 

**Figure 5:**
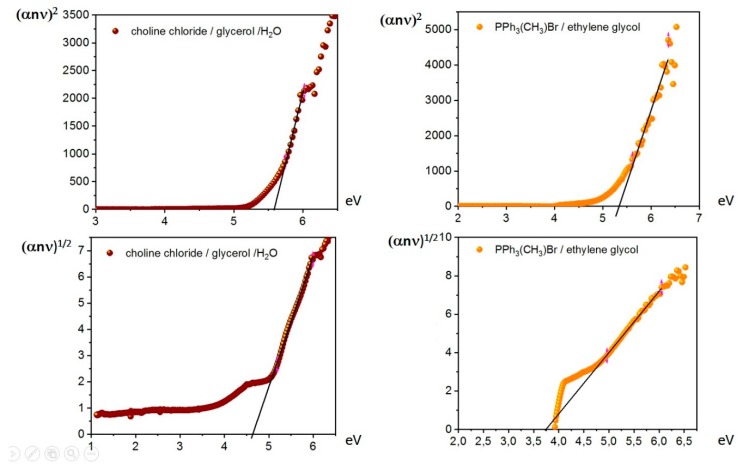
Tauc plots for (top) direct and (bottom) indirect transition of systems (left) **4** and (right) **5**.

Different behavior was observed for Systems **4** and **5**, which showed both indirect and direct transition (Figure 5).

For Systems **4** and **5**, both direct and indirect energy gaps could be determined. A summary of the conducted measurements is reported in Table 2. 

The direct band-gap energies reported in Table 2 were quite elevated for all considered systems and suggested classifying these systems as insulators (BG > 4) [29]. Only System **2** (choline chloride/imidazole) showed an energy value below 5 eV. Among systems containing triphenylmethylphosphonium bromide, System **5** was of interest, showing an indirect band gap of 3.74 eV, similar to other known semiconductors [30,31,32,33]. To date, 3.74 eV is the lowest value of an energy band gap ever reported for such kind of systems. The determination for the same system of both direct and indirect band gaps suggests the presence of different phases with different optical behavior in Systems **4** and **5**. Band-gap energy crossover composition was determined for several systems, for example, for the system of AlxGa1 - xAs [34], for BexZn1 - xTe alloy [35], for double perovskite systems [36], for MoSe_2_ and MoS_2_ [37], for Group IV semiconductor alloys [38], and for 2D van der Waals crystals [39]. Some information about the perturbation of the structural disorder of systems could be extracted from the absorption-edge region of the curves reported in the Tauc plot [40]. This is an exponential part of the curve known as the Urbach tail resulting from localized states that are extended in the band gap [40]. In this low-photon-energy area, the system follows the Urbach rule, which relates absorption coefficient and photon energy Equation (9) [41].

α = α_0_ exp(hν/Eu), (9)

where α is the absorbance coefficient, α_0_ a constant, and Eu the Urbach energy.

Urbach energy is related to temperature and is indicative of the disorder of low crystalline materials [41]. By applying the natural logarithm function to Equation (9), a linear curve was obtained Equation (10).

ln(α) = ln(α_0_) + hν/Eu. (10)

Thus, by plotting α vs. photon energy, it is possible to obtain a straight line of which the slope is the inverse of the Urbach energy. In Table 3, Eu values are reported for Systems **1**–**6**.

Looking at the data in Table 3, some trends are observable. Eu energies that are related to the disorder and defects [42] follow the order of triphenylmethylphosphonium bromide/ethylene glycol > triphenylmethylphosphonium bromide/glycerol > choline chloride/nickel sulphate, choline chloride/imidazole, choline chloride/glucose > choline chloride/glycerol. 

Concerning choline chloride-based systems, in order to perform appropriate analysis of the acquired data, we compared the results of the present study with literature data [11] (Table 4). 

The systems reported in Table 4 differ by the nature of the HBD. Some attempts at classification based on HBD acidity were reported [43]. Nevertheless, no direct correlation between pKa values of former HBDs and BG energy was observed. As a matter of fact, from the reported data in Table 4, it is possible to observe some trends. Systems containing glycolic acid and imidazole showed very similar BG energy of around 4.7 eV (Entries 1 and 2). The presence of levulinic acid, ethylene glycol, and nickel sulphate pushed BG energy between 5.0 and 5.2 eV (Entries 3–5). Finally, systems containing glucose and glycerol had the highest BG energy value (Entries 6 and 7). Looking at the chemical nature of the HBD, polyols and Lewis acids seemed to increase band-gap energy (Entries 4–6). This trend was already observed for systems containing choline chloride and CuSO_4_, or ZnCl_2_ [11], and was confirmed here. Among Bronsted acids, glycolic is more efficient in reducing the BG than levulinic (Entry 1 vs. Entry 3), revealing an important effect of the specific HBD on band-gap energy. 

Finally, as all systems reported in Table 4 contained 10 wt % of water, some considerations about its presence deserve attention. The addition of increasing water amounts in several eutectic mixtures revealed a progressive transition from a water-in-DES regime, where the eutectic mixture preserved its nanostructure, to a DES-in-water regime, where the eutectic mixture became a simple aqueous solution of dissolved species. Even if the effect of water on the hydrogen-bonding network of a DES is still debated, it is generally accepted that the representative DES reline retains interactions between its components up to water concentrations as high as 35 wt %, and a transition from a water-in-DES to a DES-in-water regime occurs only at ca. 50 wt % [44,45]. Therefore, we are confident that the addition of 10 wt % of water in all the systems of Table 4 was considerably below the maximal limit of tolerance, and molecular interactions were preserved while reducing viscosity.

An additional issue is represented by the possibility of water to act as additional HBD and to contribute to band-gap-energy determination. This topic was discussed for some of the systems reported in Table 4, and, as a general observation, the presence of 10 wt % of water did not affect band-gap energy for the system containing glycolic acid (Entry 1), and reduced the band-gap energy of systems containing levulinic acid (of 0.14 eV, Entry 3), and ethylene glycol (of 0.76 eV, Entry 4). Previously published data showed no linear behavior of water presence on band-gap energy [11].

## 3. Materials and Methods

### 3.1. Sample Preparation

Choline chloride (99%) as well as triphenylmethylphosphonium bromide (98%) have been purchased from Merk.

The HBA (choline chloride or triphenylmethylphosphonium bromide) and the selected HBD were placed in a 2 mL vial and stirred at 80 °C for 16 h. Due to the very high viscosity of Systems **1**–**3** at room temperature, 10 wt % of water was added after melting and prior to UV–VIS measurements. We also added 10 wt % of water to Sample **4** for comparison.

### 3.2. UV–VIS Analysis

UV–VIS analyses were performed on samples in pure form by employing a Hewlett Packard 845-3 UV–Visible system (HP, Palo Alto, CA, United States). Samples were analyzed in a 0.7 mL quartz cuvette.

## 4. Conclusions

Six molten systems containing choline chloride or triphenylmethylphosphonium bromide as the hydrogen bond acceptor were prepared and analyzed with UV–VIS. From the optical-absorbance data through the graphic Tauc plot method, we could determine the corresponding direct band-gap energy for each system. For the systems of choline chloride/glycerol and triphenylmethylphosphonium bromide/ethylene glycol, indirect band-gap energy was found. Analysis of data relative to direct band-gap energy, integrated with literature measurements, revealed an effect of the band-gap widening of hydrogen bond donors based on Lewis acids and polyols, and high sensitivity of band-gap energy to a specific hydrogen bond donor. Additional studies for promising systems are needed, such as a detailed characterization of band structures and the influence of a single HBD and HBA on direct or indirect transition. Nevertheless, the reported data reinforce the idea that DESs or DES-like systems can find applications as optical materials.

## Figures and Tables

**Table 1:** Composition and nomenclature of presented systems. Note: HBA, hydrogen bond acceptor; HBD, hydrogen bond donor.

System	HBA	HBD	Molar Ratio
**1**	Choline chloride	nickel sulphate	1:1^a^
**2**	Choline chloride	imidazole	3:7^a^
**3**	Choline chloride	d-(+)-glucose	2:1^a^
**4**	Choline chloride	Glycerol	1:5^a^
**5**	Triphenylmethylphosphonium bromide	ethylene glycol	1:5
**6**	Triphenylmethylphosphonium bromide	Glycerol	1:5

^a^10 wt % of water added before UV–VIS analysis.

**Table 2:** Indirect and direct band-gap energies for Systems **1**–**6**. Pearson’s coefficient of corresponding linear fitting reported in parentheses.

Entry	HBA	HBD	Indirect Band Gap (eV)	Direct Band Gap (eV)
**1**	Choline chloride	Nickel sulphate	-	5.18^a^ (0.96945)
**2**	Choline chloride	Imidazole	-	4.74^a^ (0.98835)
**3**	Choline chloride	d-(+)-glucose	-	5.85^a^ (0.9828)
**4**	Choline chloride	Glycerol	4.64^a^ (0.99756)	5.56^a^ (0.98955)
**5**	Triphenylmethylphosphonium bromide	Ethylene glycol	3.74 (0.99879)	5.34 (0.97962)
**6**	Triphenylmethylphosphonium bromide	Glycerol	-	5.23 (0.98312)

^a^10 wt % of water added before UV–VIS analysis.

**Table 3:** Urbach energies for Systems **1**–**6**.

Entry	HBA	HBD	Eu	Direct Band Gap (eV)
**1**	Choline chloride	Nickel sulphate	0.59	5.18
**2**	Choline chloride	Imidazole	0.34	4.74
**3**	Choline chloride	d-(+)-glucose	0.26	5.85
**4**	Choline chloride	Glycerol	0.13	5.56
**5**	Triphenylmethylphosphonium bromide	Ethylene glycol	1.56	5.34
**6**	Triphenylmethylphosphonium bromide	Glycerol	0.67	5.23

**Table 4:** Direct band-gap energies of choline chloride-based systems.

Entry	System	Direct Band Gap (eV)
1	Choline chloride/glycolic acid/H_2_O	4.68 [11]
2	Choline chloride/imidazole/H_2_O	4.74
3	Choline chloride/levulinic acid/H_2_O	5.08 [11]
4	Choline chloride/ethylene glycol/H_2_O	5.16 [11]
5	Choline chloride/nickel sulphate/H_2_O	5.18
6	Choline chloride/glucose/H_2_O	5.85
7	Choline chloride/glycerol/H_2_O	5.56
